# *Tribulus terrestris* Alters the Expression of Growth Differentiation Factor 9 and Bone Morphogenetic Protein 15 in Rabbit Ovaries of Mothers and F_1_ Female Offspring

**DOI:** 10.1371/journal.pone.0150400

**Published:** 2016-02-29

**Authors:** Desislava Abadjieva, Elena Kistanova

**Affiliations:** Institute of Biology and Immunology of Reproduction, Bulgarian Academy of Sciences, Sofia, Bulgaria; Institute of Zoology, Chinese Academy of Sciences, CHINA

## Abstract

Although previous research has demonstrated the key role of the oocyte-derived factors, bone morphogenetic protein (BMP) 15 and growth differentiation factor (GDF) 9, in follicular development and ovulation, there is a lack of knowledge on the impact of external factors, which females are exposed to during folliculogenesis, on their expression. The present study investigated the effect of the aphrodisiac *Tribulus terrestris* on the GDF9 and BMP15 expression in the oocytes and cumulus cells at mRNA and protein levels during folliculogenesis in two generations of female rabbits. The experiment was conducted with 28 *New Zealand* rabbits. Only the diet of the experimental mothers group was supplemented with a dry extract of *T*. *terrestris* for the 45 days prior to insemination. The expression of BMP15 and GDF9 genes in the oocytes and cumulus cells of mothers and F1 female offspring was analyzed using real-time polymerase chain reaction (RT-PCR). The localization of the GDF9 and BMP15 proteins in the ovary tissues was determined by immunohistochemical analysis. The BMP15 and GDF9 transcripts were detected in the oocytes and cumulus cells of rabbits from all groups. *T*. *terrestris* caused a decrease in the BMP15 mRNA level in the oocytes and an increase in the cumulus cells. The GDF9 mRNA level increased significantly in both oocytes and cumulus cells. The downregulated expression of BMP15 in the treated mothers’ oocytes was inherited in the F1 female offspring born to treated mothers. BMP15 and GDF9 show a clearly expressed sensitivity to the bioactive compounds of *T*. *terrestris*.

## Introduction

The plant, *Tribulus terrestris* (*TT*), is used in the industrial production of medical preparations and feed additives based on its saponin fraction [1]. The dry extract of *TT* is extremely rich in substances having potential biological significance, such as flavonoids, alkaloids [2], glycosides, and phytosteroids [3–5]. Since ancient times, *T*. *terrestris* is known as an aphrodisiac herb with stimulating effects on the androgenic metabolism in humans and male animals [6–11]. Recent studies show that this plant can also influence female reproduction and improve female sexual dysfunction [12]. Oral treatment of aqueous extracts of *TT* increases the number of growing follicles in mice, but without significant effect on the sex hormone levels [13]. The increase in egg production in Guinea fowls treated with *TT* extract report [14]. [15] showed higher numbers of yellow bodies in the ovaries of treated immature rats as an evidence of LH-like activity of *TT*, causing puberty onset. Moreover, *TT* was suggested as an alternative treatment for polycystic ovary disease. A high dose of the extract can efficiently remove ovarian cysts and resume ovarian activity [16–17]. It seems that *TT* affects folliculogenesis in females; however, limited information is available in this regard and the mechanisms underlying the action of *TT* remain unknown.

It is well known that two oocyte-specific genes, growth differentiation factor 9 (GDF9) and bone morphogeneticprotein 15 (BMP15), play a key role in the regulation of folliculogenesis in many species. These factors induce the proliferation and differentiation of the follicular cells during follicular development from the primordial stage [18–19]. In addition, they are involved in the final events of maturation and ovulation, such as cumulus cell expansion and yellow body formation [20–22]. For efficient female fertility, a precisely balanced level of BMP15 and GDF9 expression in oocytes is required [23]. These processes are controlled and coordinated by mutually dependent actions between oocytes and cumulus cells [24]. Evidence of the importance of BMP15 and GDF9 in female fertility was obtained through experiments with knockout and transgenic animals [25–27], analyses of the gene mutations in ewes and humans [28–31], as well as through in vitro experiments with oocytes-cumulus complexes [32–34]. However, there is a lack of knowledge on how external factors affect the expression of these oocyte-derived growth factors, particularly the nutritional factors that animals are exposed to during folliculogenesis.

There are only few in vivo studies available in this field. A previous study [35], showed that a 40% calorie restriction in mice led to a decline in the expression of GDF9 in the ovaries; however, a contrasting data was provided by another study [36]: no differences were observed in the GDF9 and BMP15 mRNA levels in the oocytes of ewes exposed to the high and low energy diets for two weeks before superovulation. In rabbits, a previous study [37] examined the effect of moderate and severe dietary feed restriction (21 days), followed by refeeding (8 days), on GDF-9 gene expression in mature and immature oocytes. They established an enhanced percentage of mature oocytes, accompanied by a significant increase in GDF-9-mRNA levels in oocytes after refeeding. In contrast, another study [38] did not observe any change in the mRNA levels of GDF9 and BMP15 from whole ovaries of rabbits fed on a high fat, high cholesterol diet.

Considering the pivotal role of the paracrine factors GDF9 and BMP15 in folliculogenesis from the primordial stage until ovulation, we hypothesize that the biological active compounds of *TT* affect their expression through metabolic pathways. To test this hypothesis, we aimed to evaluate the growth factors, GDF9 and BMP15, at mRNA and protein levels to determine: a) how *TT* affects folliculogenesis and ovulation rates in rabbit-mothers and b) how *TT* impacts the ovaries of F1 female offspring born to the treated mothers.

## Materials and Methods

### Experimental design for animals

The investigations were conducted with 28 female white *New Zealand* rabbits in the animal facility of the Institute of Biology and Immunology of Reproduction, Sofia, Bulgaria. The experiment started with 14 sexually mature does (mothers generation) randomly divided in the control and experimental groups of 7 females each. The animals from the experimental group were individually provided the feed additive, VemoHerb-T (VHT, producer Vemo-Ltd, Bulgaria), with their drinking water, in daily doses of 3.5 mg/kg body weight for 45 days prior to insemination. The VHT preparation contained a dry extract of *Tribulus terrestris* with the following active substances: 60% furostanol saponins, determined as protodioscin; flavonoids—10.0%; tannic compounds—tannin—10.0%. Both groups were fed with standard diet for mature rabbits. At the end of the experimental period, female rabbits from both groups were mated with one control male rabbit. The number and survivability of offspring were estimated. The animals from the parental generation were slaughtered, after weaning of the offspring, for ovary collection.

Among the female newborns (F1 female generation), two new groups were formed, control and experimental, of 7 females each to the origin from the mothers’ generation. The animals were fed standard diet and kept in the same conditions as the mothers’ generation, but without VHT additive, until puberty onset (6 months old) and were slaughtered thereafter for ovary collection.

All animals were reared and handled in accordance with the Bulgarian Veterinary Law (25/01/2011), in terms of the life conditions and welfare of experimental animals, adapted to the European Union regulation 86/609. In accordance with the Bulgarian Veterinary Law (Regulation N20 /01.11.2012 based on the Directive 2010/63/EU on the protection of animals used for scientific purposes in the Member States), only the National Ethics commission for animals can approve the use of animas for scientific purposes. The present study is one of the planned institutional projects for the period 2013–2018 and its protocol has been approved by the National Ethics commission for animals within the delivered permission of use of the animals in the experiments" (N84/04.10.2013, expiry date 04.10.2018). The responsible person for animal welfare at the Institute, working directly with the animals, is licensed by Bulgarian veterinary services for animal experimentation. The slaughtering of animals was performed after previously anesthetizing with 2% xylazine. All efforts were made to minimize suffering.

### Sample collection

Ovaries removed from the slaughtered animals were kept in PBS and brought to the laboratory immediately. One ovary from each animal was used for the collection of oocytes and cumulus cells. The cumulus-oocyte complexes (COCs) were aspirated by puncturing the follicles (diameter ≥2 mm) and collected under the stereomicroscope SMZ-10 (Nikon, Japan). Firmly compacted COCs with multilayered cumulus cells and a uniformly granulated cytoplasm were separated into oocytes and cumulus cells by repeated aspiration using a narrow-bore Pasteur pipette. Final denudation of the oocytes was performed by hyaluronidase treatment (Sigma-Aldrich; at a working concentration of 300 μg/mL), followed by washing thrice in PBS. The collected samples were stored at -80°C until RNA extraction.

### Extraction and Purification of Total RNA and First-strand cDNA Synthesis

Total RNA was isolated from cumulus cells and oocytes separately for each animal, using a commercial RNA purification kit (NORGEN, 17200, Canada). A 1-μL volume of the solution was used for a concentration test and examination of the RNA integrity with a Qubit RNA Assay Kit (Invitrogen, Q32852). The extracted RNA was kept at –70°C.

The first strand of cDNAs was synthesized by the kit “Easy Script Plus Reverse Transcriptase“(G177, abm, Canada), according to the manufacturer’s instructions using the oligo dT and random primers mix included in the kit.

### Real Time PCR

The analysis of the GDF9 and BMP15 gene expressions in oocytes and cumulus cells was performed by RT-PCR using two sets of primers, GDF9-forward primer: 5′-*AAAGACCAGCTGCAGCATCC*-3′and reverse primer: 5′-*TGGTGTGAACTGGAGAGCCA*-3′; BMP15-forward primer: 5′-*GCTGGGATCACTGGATCATT*-3′ and reverse primer: 5′- *TGTACAGGACTGGGCAATCA* -3′. All primers were commercially synthesized (Sigma-Aldrich, USA).

PCR amplification was performed under the following conditions: predenaturation at 95°C for 10 min; 40 cycles for each sample that comprised an initial denaturation step at 95°C for 5 s, annealing at 60°C for 20 s, and extension at 60°C for 30 s. Rabbit glyceraldehyde-3-phosphate dehydrogenase (GAPDH- forward primer: AAGGTCATCCACGACCACTT; GAPDH- reverse primer: AGGCCATGCCAGTGAGT; Sigma-Aldrich, USA) was used as a control gene for PCR. At the same time, negative controls (without cDNA template), with cDNA substituted for water, were set up for comparison. Each sample was run in triplicate. The cycle threshold values (CT) indicated the quantity of the target gene in each sample and the sequence of the target gene was determined in realtime using an Mx3005P QPCR system (Stratagene, LaJolla, CA, USA).

In order to correct the inter-run fluctuations, standard curves were generated for each gene by serial dilution of the mix cDNA samples (1, 1:5, 1:25, 1:125, 1:625, and 1:3125). A 2-μL volume of each concentration was used as a sample separately for the construction of GDF9, BMP15, and GAPDH gene standard curves.

### Immunohistochemistry

The second ovary of each animal was fixed with 10% formaldehyde PBS and subsequently dehydrated and embedded in paraffin. Serial sections of 5-μm thickness were cut by using a microtome type 2125RT (Leica GmbH, Germany). The histological estimation of the folliculogenesis was done by counting the number of the different classes of follicles per μm.

For the detection and analysis of GDF9 and BMP15 proteins, the immunohistochemical technique described by [39] was performed using commercial antibodies. Simultaneously, immunohistochemical-negative controls were carried out using an irrelevant IgG. Briefly, sections were blocked with 3% hydrogen peroxide for 15 min and kept in 10% donkey serum for 1 h at room temperature. The primary antibodies goat polyclonal anti-human, in working dilution GDF-9 1:100 or BMP15 1:100 (GDF9, code sc-12244; BMP15, code sc- 18337, Santa Cruz Biotechnology, Inc.; Santa Cruz, CA, U.S.A.) were used for incubation over night at 4°C. The primary antibody reaction was achieved using donkey anti-goat biotinylated secondary antibodies in a working dilution of 1:200 (code sc-2023) for 1 h, followed by avidin-biotin complex (1:1000; code PK-6100, Vector, USA) for 1 h. Finally, the binding was visualized by the addition of 3,3′-diaminobenzidine (DAB) chromogen solution (1:200; code SK-4105, Vector, USA) for 10 min. After counterstaining with hematoxylin (Vecto, USA), the sections were observed with an Olympus BX51 (Olympus, Tokyo, Japan) and images were taken with a Powershot G6, 7.1 megapixels digital camera (Canon Inc., Japan).

### Statistical Analysis

Depending on the sample number and normality of distribution, parametric and non-parametric statistics were used. For n > 30 and normal distributions, Student’s *t*-test was applied. In all other cases, the Wilcoxon rank-sum test was used to estimate the significance of results.

The data from PCR were developed by DataAssist Sft. (Ver. 3. 01, Applied Biosystems) using the ΔCt method [40]. The quantifying results of copy numbers of BMP15 and GDF9 mRNA were expressed as a mean of the ratio of target genes to the control gene—GAPDH. For the evaluation of the feed additive effect, the ΔΔCt method was applied for the comparison between the control and the experimental groups [40]. The fold change (FC) against the control group ≥ 2 and *p* value ≤ 0.05 were considered as an up-regulation; FC ≤ 0.5 and *p* ≤ 0.05 were considered as downregulation. Genes with a FC between 0.5 and 2 were considered to indicate no significant change.

The immunostaining reaction was evaluated on representative fields of the ovary slices. Immunoreactivity of GDF9 and BMP15 was assessed as a percent of the staining intensity of the positive cells inside a defined area (μm^2^), using the software program ImageJ. A *t*-test was used to compare the results between control and experimental groups. Statistical significance was set at p < 0.05.

## Results

Supplementation of VHT in the rabbit mothers’ diet led to an increase in ovary weight in both generations of female rabbits. VHT did not affect fertility; the number of offspring born was similar to that of the control group (**S1 Table**). However, the survivability of newborns of treated mothers was higher than that of the control group (**S1 Fig**). The density of the primary and secondary follicles in the ovaries of the treated mothers’ generation and the density of the preantral follicles in the ovaries of F1 females born to the treated mothers were higher than those in the controls (**S2 Table**).

**Fig 1:**
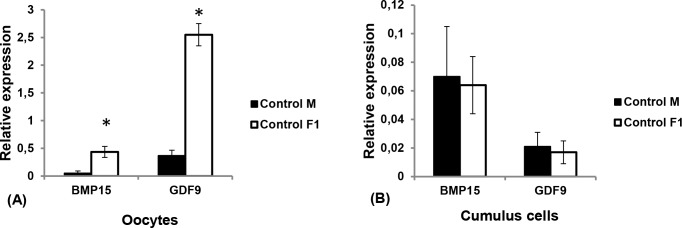
**mRNA level of GDF9 and BMP15 in the oocytes (A) and cumulus cells (B) of the female rabbits in dependence on age**. The results were obtained by comparison of the control groups from both generations–does-mothers and F1 female offspring. Total RNA was extracted from the oocytes and cumulus cells and subjected to real-time PCR to determine the mRNA levels of BMP15 and GDF9. The expression of these mRNAs was normalized to the expression of control gene GAPDH. The triplicates for each reaction were averaged. This data represent the mean ±SEM of the combined results from the analysis of seven rabbits from each group (*, P < 0.05). The significantly higher expression of BMP15 and GDF9 in the oocytes from the ovaries of F1 female compared to the mothers' generation was observed.

In the oocytes and cumulus cells collected from all does, both BMP15 and GDF9 transcripts were detected. There were the differences in the expression of the investigated genes between generations. BMP15 and GDF9 expression in the oocytes of control F1 females’ ovaries was higher than that in the control mothers’ ovaries (**Fig 1A**). A remarkable difference was observed in the expression of GDF9; the mRNA level of GDF9 in the oocytes of the F1 generations’ ovaries was several times higher than those in the mothers’ ovaries. In contrast, the expression of BMP15 and GDF9 in the cumulus cells of rabbit-mothers’ ovaries and F1 females was not significantly different (**Fig 1B**).

**Fig 2:**
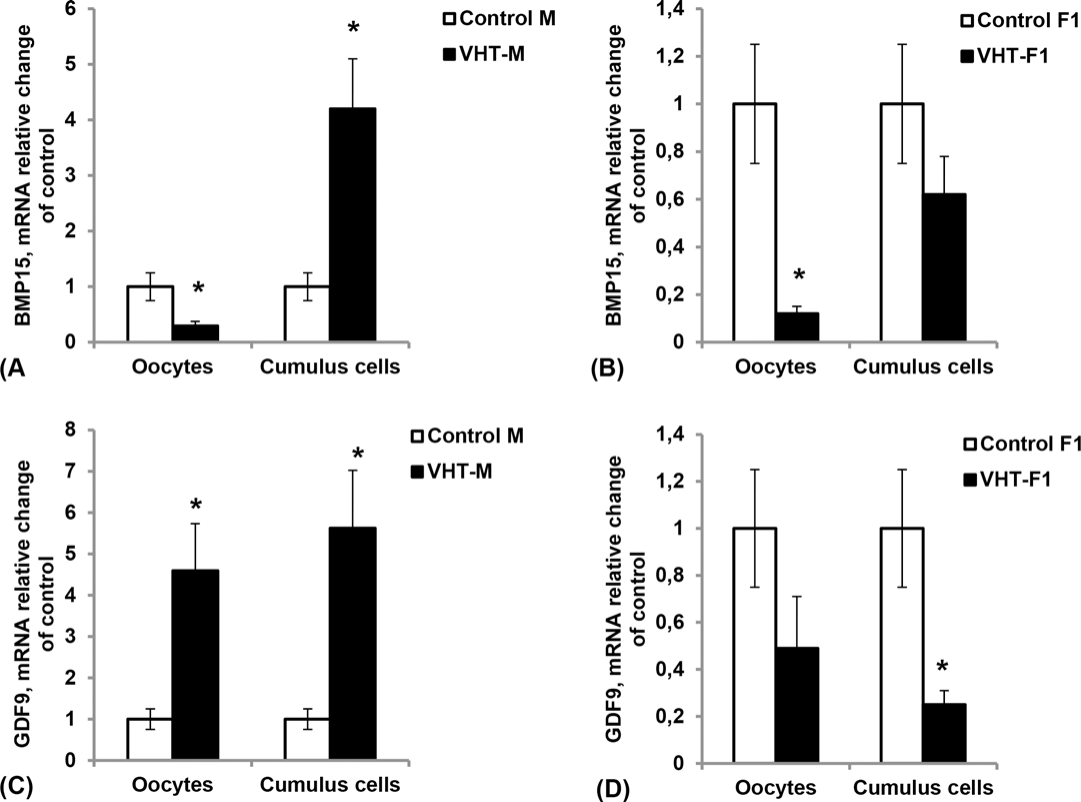
Effect of the VHT supplementation of rabbit mothers’ diet on the expression of BMP15 and GDF9 mRNA levels in the oocytes and cumulus cells of two generations. (A) and (C) mothers’ generation; (B) and (D) F1 female generation. The only experimental group of the mothers’ generation was treated with feed additive VemoHerb-T (dry extract of plant *Tribulus terrestris*, producer Vemo-Ltd) during 45 days prior to insemination. Total RNA was extracted from the oocytes and cumulus cells and subjected to real-time PCR to determine the mRNA levels of BMP15 and GDF9. The expression of these mRNAs was normalized to the expression of control gene GAPDH. Data are shown as a relative ratio (fold change—FC) of the mRNA level of these genes in the experimental groups to the controls of each generation. These data represent the mean ±SEM of the combined results from the analysis of seven rabbits from each group (*- significance P < 0.05, compared to control). *T*. *terrestris* caused a decrease in the BMP15 mRNA level in the oocytes and an increase in the cumulus cells. The GDF9 mRNA level increased significantly in both oocytes and cumulus cells. The downregulated expression of BMP15 in the treated mothers’ oocytes was inherited in the F1 female offspring born to treated mothers.

Supplementation of the rabbit-mothers’ diet with the feed additive VHT led to a decrease in the BMP15 mRNA level in the oocytes and an increase in its level in the cumulus cells (**Fig 2A**). The significant increase in GDF9 mRNA levels in both oocytes and cumulus cells was observed in the ovary of mothers treated with VHT (**Fig 2C**).

The expression level of mRNA in the investigated genes was lower in both oocytes and cumulus cells in the ovaries of F1 females’ offspring born to experimental mothers compared to the F1 females born to the controls (**Fig 2B and 2D**). We noticed down-regulation of BMP15 in oocytes of the offspring (FC = 0.12, P < 0.05) and GDF9 in cumulus cells (FC = 0.25, P < 0.05).

The direct effect of the VHT supplementation on the expression of BMP15 and GDF9 genes in the oocytes and cumulus cells of the treated mothers was several times higher than that in the F1 female offspring born to them (**Table 1**).

**Table 1:** The impact of VHT on the changes in BMP15 and GDF9 expression in both generations of female rabbits (FC_VHT-M_/ FC_VHT-F1_).

Parameters	oocytes	cumulus cells
**BMP15**	2.50	6.80
**GDF9**	9.30	22.40

FC_VHT-M_ and FC_VHT-F1 −_the fold change of gene expression relative to the control in the treated mothers and F1 female offspring born to treated mothers. The remarkable changes in the expression of the GDF9 in cumulus cells were noticed.

Both the investigated genes demonstrated sensitivity to VHT treatment. However, a more powerful impact of VHT on the GDF9 gene expression in the cumulus cells of mothers’ ovaries was observed.

The localization of BMP15 and GDF9 proteins was determined by immunohistochemistry.

**Fig 3:**
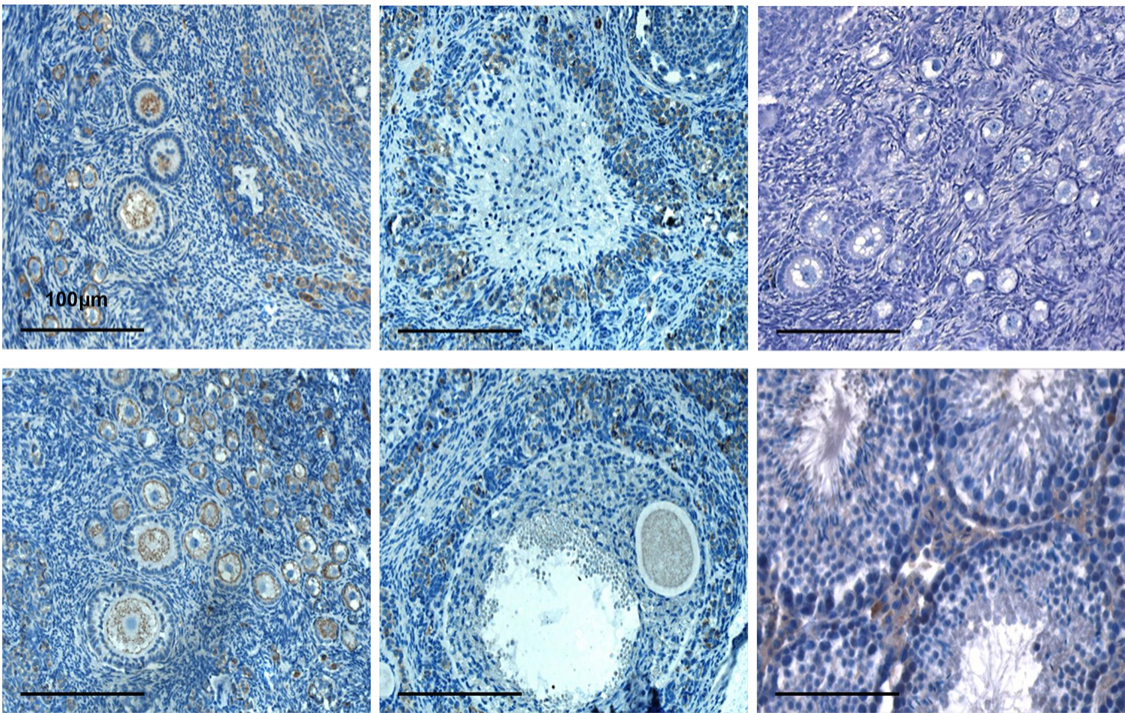
Immunohistochemical staining for BMP15 in the rabbit ovaries of the mothers and F1- female offspring generations. Localization of the protein BMP15 in: (a) oocytes of the primordial and primary follicles in the VHT-treated mothers ovary; (b) granulosa cells of the tertiary follicle in the VHT- treated mothers ovary; (c) primordial and primary follicles—negative control of antibody; (d) oocytes of primordial and primary follicles in the ovary of F1 offspring born to treated mothers; (e) oocytes and granulosa cells of the antral follicle in the ovary of F1 offspring born to treated mothers; (f) mouse testis—positive control of antibody; (g) Original magnification: (a–f) × 20.

**Fig 4:**
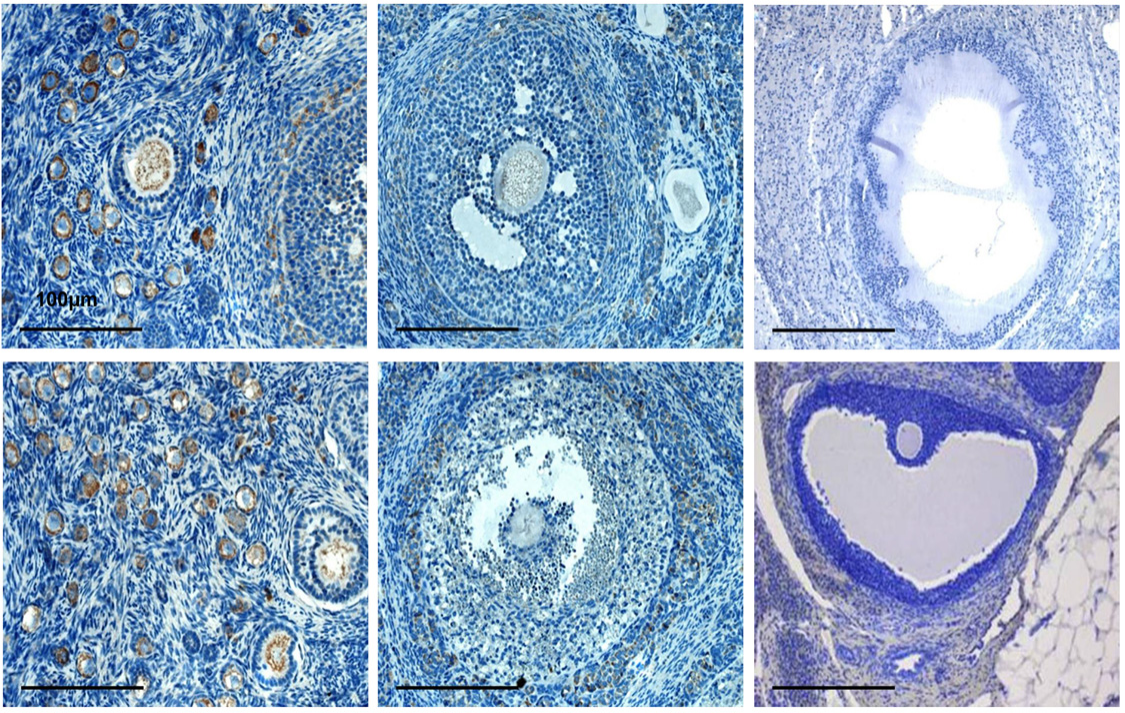
Immunohistochemical staining for GDF9 in the rabbit ovaries of the mothers and F1- female offspring generations. Localization of the protein GDF9 in: (a) oocytes of the primordial and primary follicles in the VHT-treated mothers ovary; (b) oocytes and granulosa cells of the early antral follicle in theVHT- treated mothers ovary; (c) antral follicle- negative control of antibody; (d) oocytes of primary and primordial follicles in the ovary of F1 offspring born to treated mothers; (e) oocyte and cumulus-granulosa cells of the antral follicle in the ovary of F1 offspring born to treated mothers; (f) mouse ovary—positive control of antibody; (g) Original magnification: (a–f) × 20.

Immunopositive signals of BMP15 and GDF9 were detected in the oocytes and cumulus cells of the follicles in the ovaries of mothers and F1 females’ (**Figs 3 and 4**) from both groups, control and experimental. In addition, both growth factors were detected in the theca, interstitial, and corpus lutein cells.

**Fig 5:**
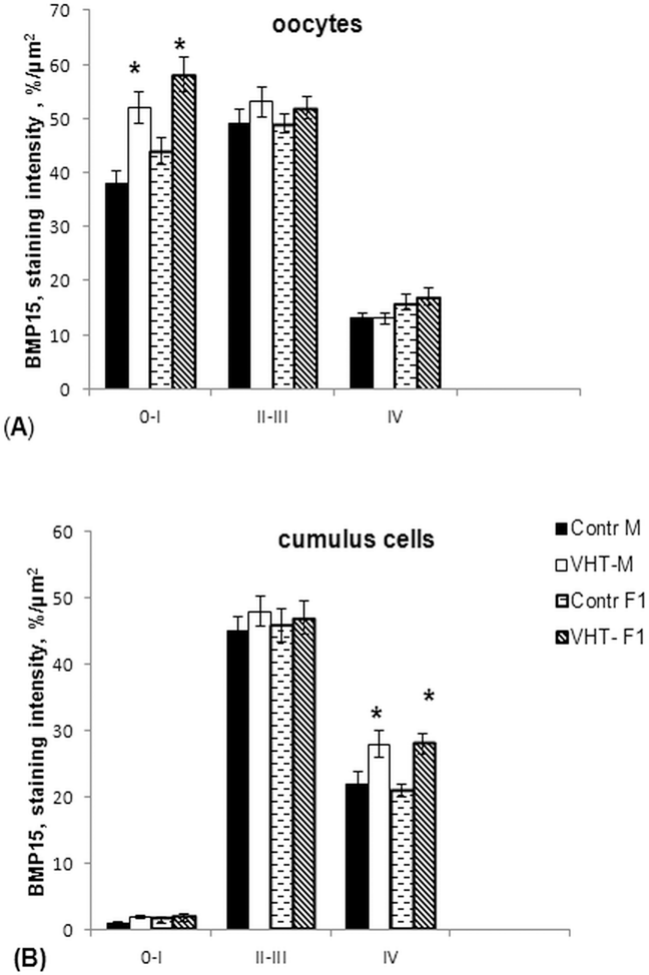
The dynamic of BMP15 protein expression in the oocytes and cumulus cells of the follicles at different stages of the development in rabbit ovaries. Control M–mothers generation; Control F1- female offspring born to the control mothers; VHT-M- VemoHerb-T treated mothers; VHT-F1- female offspring born to the treated mothers; 0-I–primordial and primary follicles; II-III–secondary and preantral follicles; IV–antral follicles. *—significant differences, p < 0.05. Protein expression was estimated by the program ImageJ based on the intensity of immunostaining reactions on the defined square. At least 5 slides per animal were analysed. Significantly intensive signals were detected in the oocytes from primordial and primary follicles in the ovaries of treated mothers as well as in the cumulus cells from the antral follicles of F1 females born to the treated mothers.

**Fig 6:**
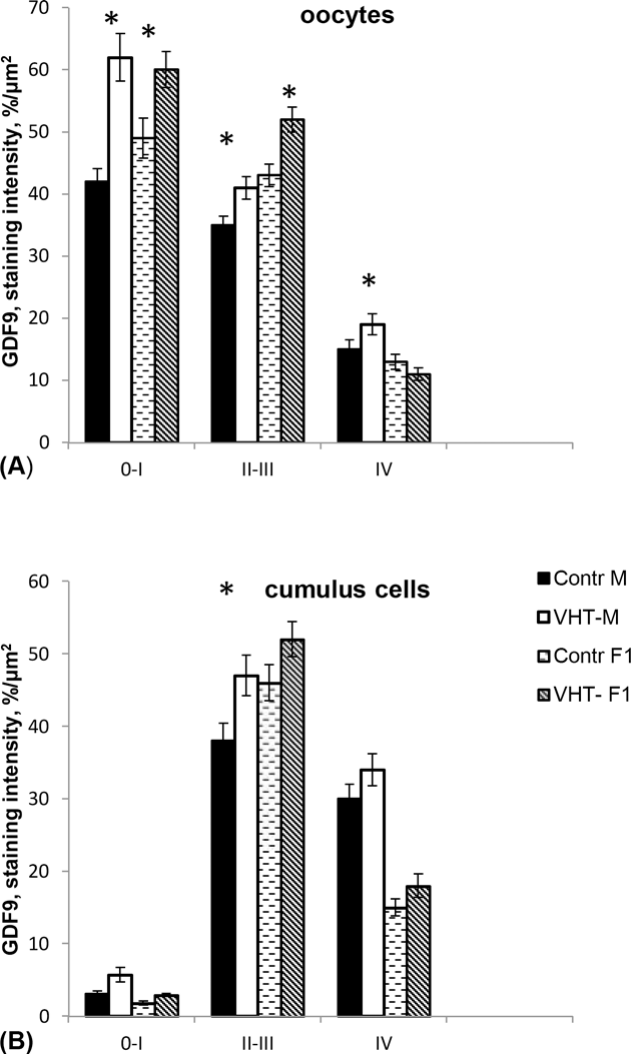
The dynamic of GDF9 protein expression in the oocytes and cumulus cells of the follicles at different stages of the development in rabbits ovaries. Control M–mothers generation (n = 7); Control F1- female offspring born to the control mothers (n = 7); VHT-M- VemoHerb-T treated mothers (n = 7); VHT-F1- female offspring born to the treated mothers (n = 7); 0-I–primordial and primary follicles; II-III–secondary and preantral follicles; IV–antral follicles. *—significant differences, p < 0.05. Protein expression was estimated by the program ImageJ based on the intensity of immunostaining reactions on the defined square. At least 5 slides per animal were analysed. Significantly intensive signals were detected in the oocytes from primordial, primary and secondary follicles in the ovaries of threated mothers as well as in the oocytes from secondary follicles of F1 females born to the treated mothers.

The computer estimated immune staining intensity of the investigated proteins had shown the most intensive signals of BMP15 (**Fig 5**) and GDF9 (**Fig 6**) in the oocytes from primordial and primary follicles and the lowest in the oocytes from antral follicles in all groups. Significantly higher signals for both proteins were observed in the primary and secondary oocytes from ovaries of the mothers treated with VHT. The increased secretion of GDF9 in the oocytes from antral follicles of treated mothers’ ovaries corresponded with the results of mRNA level changes obtained by PCR, whereas the results related to the BMP15 signals in the oocytes are somewhat contradictory. Although the level of BMP15 mRNA in the oocytes of treated mothers decreased, there were no differences in the intensity of protein signals compared to the control oocytes. It is likely that a reverse of the protein BMP15 from cumulus cells to oocytes occurred due to a deficit in mRNA production in the oocytes. Thus, the BMP15 protein expression was significantly higher in the cumulus cells of the antral follicles in both experimental groups F0 mothers and F1 female offspring generations (**Fig 5**).

In the cumulus cells, the intensity of BMP15 and GDF9 signals increased from primary to secondary and preantral follicles and decreased again to antral follicles. The more intense GDF9-signals were detected in the cumulus cells from the secondary follicles of the treated mothers (**Fig 6**).

## Discussion

Previous studies have shown that GDF9 and BMP15 have distinct effects on the reproductive physiology in a species-specific manner [41]. Considering this, it is very important to collect data on BMP15 and GDF9 actions in the ovaries of different animal species. To our knowledge, our study was the first *in vivo* study describing the changes in the expression of the growth factors BMP15 and GDF9 at mRNA and protein level in the follicular structures of female rabbits in two generations–mothers and F1 female offspring–as a result of supplementation of dry extract of *T*. *terrestris* to mothers’ diet 45 days prior to insemination. The obtained data demonstrated the clearly expressed sensitivity of the BMP15 and GDF9 to the bioactive compounds of *T*. *terrestris*.

*T*. *terrestris* is one of the most popular phyto-aphrodisiac for male reproduction. Although the long-term use of this plant improves sexual performance in males, there is no consensus on the exact mechanisms of *TT* action in the organism. It is believed that steroidal saponins present in *TT* extracts can enhance the endogenous androgen production by increasing luteinizing hormone release from the pituitary gland [42]. Another hypothesis proposes that *TT* bioactive compounds might be enzymatically converted into weak androgens such as dehydroepiandrosterone, which could be converted into more potent androgens such as testosterone or estradiol in the gonads [43]. The potential application of *TT* as an alternative therapy for the sexual dysfunction in women [12] as well as the evidence of its ability to remove ovarian cysts [16] has led to increased research on this plant focusing on female reproduction.

Our results show the stimulating effect of the additive VemoHerb-T on the weight of rabbit mothers’ ovary and weight of the offspring. Similar results were observed in mice [13] and in Guinea fowls [14] treated with the extract of *TT*. Moreover, the treated mother does in our experiment expressed more pronounced maternal instincts than the controls. They more actively prepared the nests and took thorough care of the pups. The Kaplan-Meier analysis confirmed a higher survivability of newborns to experimental mothers. Considering that such maternal behavioral reactions are related to the action of estrogens on the central nervous system [44], our results indicate an enhanced level of estrogens in the ovary of treated mothers. Our results are consistent with a previous study [42], which demonstrates an increase in the luteinizing hormone level in female rats treated with *TT*. The LH-like effect of *T*. *terrestris* on rats’ ovaries has been demonstrated in a previous study [15]. It is known that LH can stimulate estradiol production in the ovary by the activation of the aromatase pathway [45]. An enhanced level of estradiol was reported in women who used the preparation Tribestan (pills containing the dry extract of *TT*); despite the suppression of FSH production, the secretion of estradiol and progesterone did not decrease in women treated with Tribestan [46].

However, it is impossible to explain all morphological changes in the ovaries of experimental animals, particularly the increase in the density of secondary follicles, by the hormonal changes only, because at this stage they are not sensitive to hormones. Similar to the present study, an enhanced number of secondary follicles in the ovaries of *TT* treated rat was found in a previous study [15]. These results can be explained on the basis of our data, which demonstrated the changes in the expression of growth factors BMP15 and GDF9 in the oocytes and cumulus cells under *TT* treatment.

Currently existing literature related to rodents mainly provides information on the expression of GDF9 and BMP15 genes in the oocytes [47, 26]. In our in vivo experiments, we established new insights that the transcripts of BMP15 and GDF9 were detected in both oocytes and cumulus cells in the ovaries of all adult and young rabbits. Their expression was higher in the oocytes of young animals, whereas the expression in the cumulus cells exhibited no significant difference between the young and adults. These results correspond with the results of previous studies on the age-associated profiles of BMP15 and GDF9. Significant BMP15 gene expression in rabbit ovaries from 14 d post parturition were observed, when primordial follicles were clearly detected [48]. The highest level was measured at 60 d post parturition and a lower level was detected in adult animals. Similar results were obtained during the investigation of gene profiles in the oocytes from young and adult mice [49,35]. Our results confirmed the findings of a previous study [50] that GDF9 expression is higher in the oocytes of young calves than in adult cows. However, our results differ from the results of that study [50] in terms of age-associated BMP15 expression in bovine oocytes and cumulus cells.

We established the presence of BMP15 and GDF9 proteins in the follicles at all stages of development as well as in the interstitial and corpus lutein cells, which strongly suggest their pivotal role in the whole process of folliculogenesis in rabbits and confirms the previous investigations in other rodents [27, 32].

In the present study, the alteration in the expression of the BMP15 and GDF9 genes, as a result of the supplementation of feed additive VHT to the mothers’ diet, indicated their high sensitivity to metabolic changes in the ovaries. These findings are consistent with those of previous studies [35, 37], in which the calorie restriction in mice, as well as the feed restriction and refeeding in rabbits lead to changes in GDF9 expression in the ovaries. In our study, *T*. *terrestris* caused the alteration of mRNA levels of both genes, but in different ways. Although we noticed a downregulation of BMP15 in the oocytes of treated mothers, we did not observe any failure in their fertility. This corresponds with the conclusion of [32] that in rodents, BMP15 is only partially required for ovulation and fertilization. Nevertheless, the observed increase in BMP15 mRNA level in the cumulus cells possibly compensates the lack of BMP15 expression in the oocytes.

Additionally, we found a significant upregulation of the GDF9 gene in the oocytes and cumulus cells of treated mothers. GDF9 is the closest homolog to BMP15 (known as GDF9B). Both bind to the same type II receptor, BMPRIIB, which is essential for GDF9/BMP15 signaling in granulosa cells [51, 19] and show synergism or have similar activity during folliculogenesis [52]. Moreover, GDF9 supports embryo development and fetal viability [53]. It is likely that these biological functions of GDF9 facilitated increased fertility in our experimental animals, despite the decreased level of BMP15 mRNA in the oocytes.

The morphological changes in the treated rabbits’ ovaries followed the changes in the expression of growth factors. There is substantial evidence in several mammalian species that GDF9 is essential for the early stages of follicle development [52]. The increased level of GDF9 in our study ensured a more active involvement of the ovarian reserve in the development. We observed a higher density of primary and secondary follicles in the ovary of the mothers treated with *TT*. In addition, the diameter of secondary and preantral follicles was higher than that in controls (unpublished data). This is likely due to the ability of GDF9 to regulate granulosa cell mitosis through both, Smad-dependent and independent signaling pathways [54].

To our knowledge, this is the first study to demonstrate that *T*. *terrestris* causes a significant increase in GDF9 at the mRNA and protein levels in the oocytes and cumulus cells of rabbits. These results provide additional value to the explanation of the therapeutic effect of *TT* described in previous studies [12, 16] in females, because the lack of GDF9 leads to several types of ovarian disorders. It was reported that ovaries from 25- to 31-week-old female GDF9 null mice contain either single unilateral or bilateral ovarian follicular cysts, lined by several layers of flattened granulosa cells [25]. In addition, research on ovary tissues from women with polycystic ovary syndrome (PCOS) revealed that GDF9 mRNA expression is substantially delayed and reduced during the growth and differentiation phase [55] and cannot reach the normal level even after ovarian stimulation, which is the premise for impaired oocyte quality and developmental competence in PCOS females [56]. The increased level of GDF9 mRNA in the ovaries of animals treated with *TT* should be the main reason for the success of *TT* in removing ovarian cysts [16]. Additionally, the GDF9 acting in theca cells regulates androgen production and subsequently affects granulosa cells in different ways–by facilitating granulosa cell estrogen production from androgen aromatization, or by ensuring direct effects of androgens on granulosa cell growth through the androgen receptor that is expressed in granulosa cells [34, 52].

If we accept the mainly discussed hypothesis that *TT* acts like the LH-hormone, a high level of GDF9 is also essential for modulating the LH receptors to ensure an adequate reaction to the hormone, because the oocyte-secreted factors, GDF9 and BMP15, have an effect on FSH and LH through the expression of their receptors on target cells [57–59]. Therefore, the upregulation of GDF9 in the oocytes and cumulus cells caused by *TT* explains the consequences of *TT* action. However, the exact mechanism underlying the increase in GDF9 level remains unclear and requires further investigations.

The analysis of mRNA levels of BMP15 and GDF9 in the oocytes and cumulus cells of F1 female offspring showed heritable changes in the BMP15 gene function. The down regulation of this gene was expressed also in the oocytes of F1 females born to treated mothers. Our results strongly suggest that rabbit’s oocytes are susceptible to epigenetic modulations during folliculogenesis and that the bioactive supplement VHT should be one of the factors causing this transgenerational epigenetic effect related to BMP15. Epigenetic processes can occur due to the direct exposure to external factors at critical periods, particularly during sensitive developmental windows for the germ cells, such as folliculogenesis [60]. Nutrition, as an environmental factor, affects oocytes. Therefore, changes in the environment surrounding oocytes can alter the pattern of genes expressed by these structures, with consequences for both immediate and longer-term development [61]. Despite the fact that rabbits belong to a group of mammals that do not ovulate spontaneously, it is known that follicles develop and regress in cycles of 15−16 days. It seems that the exposure of the rabbit mothers to the bioactive supplement *TT* during 45 days of the periconceptual period was sufficient to induce changes in the oocytes-secreted gene BMP15. It is likely that the pathways of *TT* action on the investigated growth factors are different. The upregulation of GDF9 mRNA in the oocytes and cumulus cells stimulated by *TT* was not inherited in F1 females; moreover, we observed a lower level of GDF9 mRNA in the oocytes and cumulus cells of these animals compared to the F1 females born to the control mothers.

## Conclusions

The present study provides for the first time the evidence for the stimulating effect of *TT* on the expression of GDF9 and BMP15 at mRNA and protein levels in the oocytes and cumulus cells of rabbits in two generations. The results indicate that these growth factors are sensitive to the bioactive compounds of *TT* in different ways at different follicle structures. At the same time, GDF9 and BMP15 showed their interchangeability in rabbits. Therefore, overexpression of GDF9 in the case of BMP15 suppression ensured normal development and ovulation of the follicles and high fertility at the first insemination after the treatment.

The present study provides insights into the therapeutic effect of the plant *T*. *terrestris* on female reproduction at the molecular level. Further studies are required to elucidate the exact pathways of *TT* action on growth factors expression, as well as on the epigenetic mechanisms of the influence of *TT* on F1 female offspring.

## Supporting Information

S1 Table Effect of VHT supplementation to the mothers’ diet on reproductive parameters. The experimental group of mothers was treated with the feed additive VemoHerb-T (dry extract of the plant *Tribulus terrestris*, producer Vemo-Ltd) 45 days prior to insemination. Both groups were mated with the same male rabbit. Data are presented as a mean ± SEM; Wilcoxon rank-sum test was used for the significance consideration.
(DOCX)

S2 Table Density of different class follicles in the ovaries of control and experimental rabbits. Serial 5-μm sections in 25 μm depth of each ovary were analyzed. Data are presented as mean ±SEM, *t*-test was applied, and the differences were considered significant at p < 0.05.
(DOCX)

S1 Fig Kaplan-Meier analysis of the F1 offspring survivability born to control and to mothers’ groups treated with VHT. The experimental group of mothers was treated with the feed additive VemoHerb-T (dry extract of plant the *Tribulus terrestris*, producer Vemo-Ltd) 45 days prior to insemination. Both groups were mated with the same male rabbit. A significantly higher survivability of F1 offspring born to experimental mothers was observed.
(TIF)
