# Supplementary material for: Tribulus terrestris Alters the Expression of Growth Differentiation Factor 9 and Bone Morphogenetic Protein 15 in Rabbit Ovaries of Mothers and F1 Female Offspring
Source: PLoS One. 2016 Feb 29;11(2):e0150400. doi: 10.1371/journal.pone.0150400 (PMC4771171; doi:10.1371/journal.pone.0150400)
Supplement: S1 Table — The experimental group of mothers was treated with the feed additive VemoHerb-T (dry extract of the plant Tribulus terrestris, producer Vemo-Ltd) 45 days prior to insemination. Both groups were mated with the same male rabbit. Data are presented as a mean ± SEM; Wilcoxon rank-sum test was used for the significance consideration. (DOCX) [file pone.0150400.s001.docx]

**S1 Table.** **Effect of VHT supplementation to the rabbit mothers’ diet on the reproductive parameters.**

| **Parameters** | **Treated with VHT (n=7)** | **Control**  **(n=7)** | **Р** |
| --- | --- | --- | --- |
| **Mothers**  **ovaries weight, (g)** | 0.211±0.007 | 0.182±0.004 | 0,02 |
| **Gestation period (days)** | 31.0±1.12 | 30.86±0.64 | - |
| **Number of newborns/per doe** | 7.14±0.86 | 6.29±0.84 | - |
| **Number of alive newborns at 14 day post/partum** | 6.14±1.16 | 2.29±0.71 | 0.005 |
| **Live body weight at 20 day post/partum (g)** | 314.90±20.31 | 254.40±15.70 | 0.02 |
| **F1 females**  **ovaries weight, (g)** | 0.137±0.0017 | 0.117±0.0015 | 0.02 |
