# Supplementary material for: Tribulus terrestris Alters the Expression of Growth Differentiation Factor 9 and Bone Morphogenetic Protein 15 in Rabbit Ovaries of Mothers and F1 Female Offspring
Source: PLoS One. 2016 Feb 29;11(2):e0150400. doi: 10.1371/journal.pone.0150400 (PMC4771171; doi:10.1371/journal.pone.0150400)
Supplement: S2 Table — Serial 5-μm sections in 25 μm depth of each ovary were analyzed. Data are presented as mean ±SEM, t-test was applied, and the differences were considered significant at p < 0.05. (DOCX) [file pone.0150400.s002.docx]

**S 2 Table. Density of the different classes' follicles in the ovaries of the control and experimental rabbits**

| **Groups** | **Density of follicles (N*10^-4^/µm^2^)** | | | |
| --- | --- | --- | --- | --- |
|  | Primary fol.  (І) | Secondary fol. (ІІ) | Preantral fol.  (ІІІ) | Antral fol.  (ІV) |
| **Does-mothers generation** | | | | |
| **Control**  **(n=7x5)** | 0.30х10^-4^  ±0.18 | 0.13х10^-4^  ±0.08 | 0.13х10^-4^  ±0.08 | 0.08х10^-4^  ±0.04 |
| **Treated with TT (n=7x5)** | 0.58х10^-4^  ±0.28 | 0.21х10^-4^  ±0.05 | 0.16х10^-4^  ±0.07 | 0.08х10^-4^  ±0.04 |
| **Р to the control** | 0.046 | 0.044 | 0.47 | 0.71 |
| **F1 female offspring generation ( without treatment of *TT*)** | | | | |
| **Born to control**  **mothers (n=7x5)** | 0.42х10^-4^  ±0.038 | 0.24х10^-4^  ±0.031 | 0.101х10^-4^  ±0.013 | 0.05х10^-4^  ±0.01 |
| **Born to treated**  **mothers (n=7x5)** | 0.41х10^-4^  ±0.038 | 0.25х10^-4^  ±0.02 | 0.135х10^-4^  ±0.019 | 0.05х10^-4^  ±0.008 |
| **Р to the control** | 0.63 | 0.49 | 0.002 | 0.69 |
