# Supplementary figures and images for: Tribulus terrestris Alters the Expression of Growth Differentiation Factor 9 and Bone Morphogenetic Protein 15 in Rabbit Ovaries of Mothers and F1 Female Offspring
Source: PLoS One. 2016 Feb 29;11(2):e0150400. doi: 10.1371/journal.pone.0150400 (PMC4771171; doi:10.1371/journal.pone.0150400)

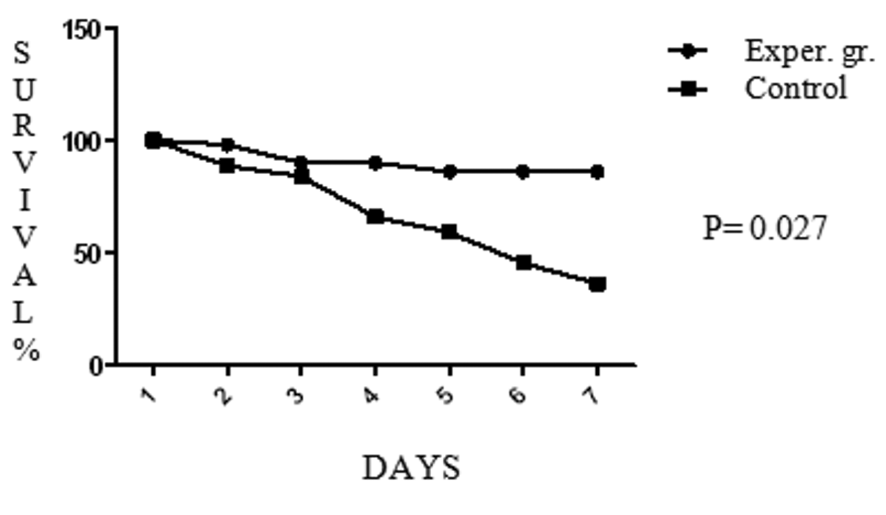

Supplement: S1 Fig — The experimental group of mothers was treated with the feed additive VemoHerb-T (dry extract of plant the Tribulus terrestris, producer Vemo-Ltd) 45 days prior to insemination. Both groups were mated with the same male rabbit. A significantly higher survivability of F1 offspring born to experimental mothers was observed. (TIF) [file pone.0150400.s003.tif]
